# Association between Socioeconomic Status, Physical Health and Need for Long-Term Care among the Chinese Elderly

**DOI:** 10.3390/ijerph16122124

**Published:** 2019-06-15

**Authors:** Fanlei Kong, Lingzhong Xu, Mei Kong, Shixue Li, Chengchao Zhou, Jianghua Zhang, Bin Ai

**Affiliations:** 1 NHC Key Laboratory of Health Economics and Policy Research, School of Public Health, Shandong University, 44 Wenhuaxi Road, Jinan 250012, China; kongfanlei@msn.com (F.K.); shixueli@sdu.edu.cn (S.L.); zhouchengchao@sdu.edu.cn (C.Z.); 2 Collaborative Innovation Center of Social Risks Governance in Health, School of Public health, Fudan University, Shanghai 200032, China; 3 Research Center of Economics and Resource Management, Beijing Normal University, Beijing 100875, China; kongmei007@126.com; 4 School of Management, Shandong University, Jinan 250100, China; zhangjianghua@sdu.edu.cn; 5 School of Ethnology and Sociology, Minzu University of China, Beijing 100081, China; aibin62@hotmail.com

**Keywords:** socioeconomic status, physical health, need for long-term care, the Chinese elderly, structural equation modeling

## Abstract

The aim of this study was to explore the relationship between socioeconomic status (SES), physical health and the need for long-term care (NLTC) of the Chinese elderly, and further, to provide evidence-based advice for establishing an LTC system in China. A cross-sectional survey was conducted in Shandong Province, China in 2017 by using multi-stage random sampling method. Data were collected from elderly individuals aged 60 years and older by self-designed questionnaires through face-to face interviews. A total of 7070 participants were finally included in the database (40.3% male, 59.7% female). Chi-square test analysis and structural equation modeling (SEM) were conducted to clarify the association between SES, physical health and NLTC among the Chinese elderly men and women in Shandong Province. The results of the SEM analysis showed that physical health exerted a strong and negative effect on the NLTC for both genders, with a slightly stronger effect found among the elderly men. SES was found to be significantly and negatively related to the NLTC among the elderly women, while no statistical significance was found for the association between SES and NLTC for elderly men. A significant and positive association between SES and physical health was observed among the elderly men and women, with a slightly stronger effect among the elderly women. Implications for lowering the NLTC and developing an LTC system were addressed based on the findings above.

## 1. Background

With a decline in fertility and mortality and an increase in life expectancy in the past decades, population aging—the continuous raise of the percentage of the population in the old age group—is accelerating globally. The total world population in 1960 was 3033 million, with 5% of population being older adults aged 65 years old and over [1]. While the total population of the world in 2019 is 7715 million, elderly people aged 65 and older constitutes 9% of the total population [2]. It is projected that there will be a world population of 9772 million in 2050, with 16% of individuals being ages 65 or older. Meanwhile, population aging will develop to varied extents in different countries. The percentage of the elderly people aged 65 and older in the more-developed countries is projected to reach 27% in 2050, up from 18% currently, while the percentage of older adults in less-developed countries is projected to double to 14% by 2050 [3]. As the largest developing country in the world, China has also experienced fast population aging in the past decades. At the end of 2018, there were 249.49 million elderly people aged 60 and more in China, comprising 17.9% of the total Chinese population [4]. Projections indicates that there will be 487 million older adults in China in 2050, accounting for 34.9% of the Chinese population [5].

Many previous studies have explored the relationship between physical health and the need for long-term care (NLTC), and have consistently shown physical health to be associated with NLTC [6]. Individuals suffering from stroke, dementia, lower limb fracture, or advanced tumors [7] are more likely to receive formal care. Moreover, those with specific comorbidities [8], including vascular dementia, incontinence, fall, hip fracture, and a number of comorbidities [9] have more opportunity to enter LTC facilities. As for the elderly in LTC institutions, a large proportion lead a sedentary lifestyle and encounter barriers to physical activities. A previous study showed that older adults in a Tai Chi (a traditional Chinese physical activity) group using wheelchairs recorded significantly higher scores on physical health than those in a control group not doing Tai Chi [10]. Qigong, which is another traditional Chinese physical exercise was also found to be a suitable daily activity for old residents in LTC facilities, and it may help with blood pressure control [11].

Existing studies show that socioeconomic status (SES) is negatively associated with NLTC [12]. Indicators of SES, such as working status, household wealth and poverty status, are related to the daily care needs [13]. Specifically, education was found to be significantly associated with the intention to use LTC facilities among the Korean elderly born between 1945 and 1954 [14]. People of both genders with low income were shown to be more likely to be functionally dependent, and use more inpatient and health care services [15]. In terms of career, a few differences in the total or public LTC use between different occupational classes were found, and those with upper non-manual jobs were found to use more private LTC services than housewives or those with lower non-manual or skilled manual jobs [16]. Besides the research above, unmet health care needs have also been found to be significantly related to SES, since more unmet care needs are reported by those in the poorer wealth status [17].

Existing research supports the widely held conclusion that SES is correlated with physical health [18,19]. Lower SES generally predicts a great severity of sleep disturbances [20], a higher incidence of mild traumatic brain injury [21], a lower score on The World Health Organization Quality of Life (WHOQOL) [22], more disability, pain catastrophizing and worse functional capacity [23]. Regarding education, lower education has been shown to be associated with a higher prevalence of physical and mental health comorbidity [24], lower scores on health related quality of life (HRQOL) [25], and higher risk for malnutrition among Māori individuals in New Zealand [26]. As for income, for both genders, low income was found to be related to a higher prevalence of diabetes [27], and a great likelihood of being functionally dependent [15]. Long-term mortality rates of stroke survivors [28], waist circumference, and body mass index [29] were clarified to be higher among those living in lower SES neighborhoods. As a close concept to SES, socioeconomic position (SEP) was also found to be a fundamental cause of diseases among African–Americans [18], and a lower subjective social status (SSS) was related to worse initial memory decline [30].

Previous studies have ever investigated the association between physical health and NLTC, between SES and NLTC, and between SES and physical health. However, no study has explored the relationship between SES, physical health and NLTC simultaneously by using Structural Equation Modelling (SEM) among the Chinese elderly in the traditional living area of the Chinese population. The current study aims to fill this research gap, and further clarify whether a gender difference exists in the relationship between SES, physical health and NLTC among Chinese seniors in Shandong Province.

## 2. Methods

### 2.1. Introduction of Study Location

Shandong Province, which lies between Beijing and Shanghai, is a coastal province and belongs to the Eastern China Region. The administrative region of Shandong Province includes 17 prefecture-level cities, 137 counties and 1826 towns. Shandong Province is important for Chinese history and civilization, and served as a pivotal cultural and religious site for Confucianism, Chinese Buddhism and Taoism [31]. In 2018, the gross domestic products (GDP) of Shandong Province was 7646.97 billion RMB (≈1139.94 billion US Dollars), an increase of 6.4% from the year of 2017. The per capita GDP was 76267 RMB (≈11,369 US Dollars) in 2018. The total number of residents in Shandong Province in 2018 was 100,472.4 thousand, of which elderly people older than 65 years accounted for 15.04% [32]. Since 2017, Shandong Province has ranked first in China for the total number of the elderly, with 3.81 million more elderly people than Jiangsu Province, which ranked in second place [33].

### 2.2. Data Collection and the Research Subjects

Data was collected by face-to-face interviews where a structured questionnaire was carried out by Shandong University as part of the 2017 Survey of the Shandong Elderly Family Health Service. To achieve the representation of the whole population in Shandong Province, the multi-stage random sampling method was used to select the participants by three steps as some previous studies did [34,35]. Firstly, 6 counties were selected from 137 counties as the primary sampling units (PSUs) in Shandong Province in consideraton of the GDP and the geographic location. These 6 counties belong to eastern regions (2 counties), central regions (2 counties), and western regions (2 counties) of Shandong Province. These 6 counties also include urban areas (3 counties) and rural areas (3 counties). Secondly, 18 villages in the rural areas (3 counties) and 18 communities in the urban and suburban areas (3 counties) were chosen from each PSU as the secondary sampling units (SSUs). Thirdly, based on the roster of elderly residents in each selected site provided by the local government, an average of 66 older adults were stratified and randomly chose from each SSU to constitute the total sample. In this way, the participants from different economic development level (such as the rural and urban area) and different geographic location (eastern, central, and western) in Shandong Province were included in our database and analysis. Thirty-minute face-to-face interviews were carried out at every subject’s home by a doctoral/master student from Shandong University.

Those aged 60 and above with local household registrations at the time of the interview were eligible for our study. A total of 7088 older adults were initially chosen and interviewed. However, 18 of them were excluded from the sample due to obvious logical errors in the questionnaire or uncompleted questionnaires. A total of 7070 elderly individuals were eventually included in the database.

### 2.3. Measurements

Table 1 illstrates the definitions and measurements (including the corresponding opinions) of the related variables in the curent study. SES is a broad concept that is used to describe an individual’s position in society. It includes different aspects, such as education level, job status, income, wealth status, and deprivation [36,37]. Education level, annual income and job before retire were used to assess SES in this study. Education has played a central role when analyzing the SES-health gradient ever since educational attainment was employed as the primary indicator of SES by Kitagawa and Hauser in 1973 [38]. For this study, education level was evaluated by asking the participants “which level of education did you finish?” with four choices: (1) Illiteracy, (2) Graduate from Elementary School, (3) Graduate from Junior Middle School, and (4) Graduate from Senior Middle School and above. Low income is not only associated with many other factors contributing to poor health, but also could be independently related to an individual’s health status [39]. Researchers treat income as ”one of the most profound influences on mortality” [40]. In this study, the annual income was measured by asking about the subjects’ incomes in the last year with the following five choices: (1) 0–2200 RMB (USD = 328), (2) 2201–3300 RMB (USD = 492), (3) 3301–6200 RMB (USD = 924), (4) 6201–21,400 RMB (USD = 3190) and (5) more than 21,400 RMB (USD = 3190). Factors related to job status, such as job insecurity [41], unemployment [42], high job strain [43], and high level occupational physical activity [44], were clarified to be risk factors of the health outcomes. Job before retire was used to assess SES in this study, and was evaluated by the following eight categories: (1) professionals/technical, (2) leaders of government/public institution, (3) clerks or staff, (4) businessman/commercial servants, (5) farming/forestry/fishing industry, (6) transportation industry/manual workers, (7) privately/individually-owned business, and (8) other jobs.

The concept of physical health mainly relates to the efficient functioning of the body and its systems, and includes the physical capacity to perform tasks and physical fitness [45]. In this study, physical health was evaluated by the following four questions: (1) “How was your physical health status last month (physical health last month)?”, (2) “How many chronic diseases did you have during the last six months (number of chronic diseases)?”, (3) “Did you pain or discomfort last month (pain/discomfort)?”, and (4) “Were you sick or injured during the last two weeks (sick/injured last 2 weeks)”.

A national long-term care insurance system has not yet been founded in China, which made the assessment of NLTC complicated. In this study, we used the Activities of Daily Living score (ADL score) as well as the three following questions to evaluate the NLTC status of the older adults: (1) “do you need long-term care (LTCN)?” (2) “How about your hearing ability (hearing loss)?” and (3) “how many times have you used the inpatient care services (times of hospitalization)”. The ADL score is based on the ADL scale, which was employed to evaluate the daily living ability of the older adults by 14 items. In this study, the ADL scale is consisted by the Physical Self-Maintenance Scale (PSMS) and the Instrumental Activities of Daily Living Scale (IADL) [46]. The 6 items on the PSMS includes going to the toilet, eating, dressing, grooming, walking and bathing. The 8 items on the IADL included telephoning, shopping, food preparation, housekeeping, laundering, using transportation, taking medicine and financial behavior. The interviewer circled the most suitable options (1 = can do it by myself, 2 = difficult, 3 = needs help, 4 = can not do it) according to their observations of participants and their reply during the survey. If a participant could not reply or could not express themselves logically correct (for example, due to dementia or aphasia), the answer were based on the observation of their family members, nursing staff or other insiders. The minimum total score of the ADL scale was 14 points; this was given if all answer to the 14 questions were the first option. The maximum total score is 56, this would happen if all 14 questions were answered with the fourth option. When 2 or more items were equals or more than 3 points, or the total score was more than 22 points, obvious dysfunction of the corresponding subject was implied.

### 2.4. Statistical Analysis Approach

A descriptive statistics analysis was conducted to describe the socio-demographic characteristics of the subjects. The Chi-square test was employed to clarify the gender differences in the socio-demographic characteristics, SES indicators, physical health indicators, and NLTC indicators. Statistical significance was set at *p* ≤ 0.001. All data analyses above were performed by using IBM SPSS Statistics for Windows (IBM Corp. Released 2017, Version 25.0; IBM Corp., Armonk, NY, USA).

SEM was employed to determine the association between SES, physical health and NLTC of the Chinese older adults in Shandong Province. The IBM SPSS Amos version 25.0 statistical software package for Windows was employed to perform the SEM and obtain generalized least squares estimates of the model parameters and to calculate the fitness indexes of the model. There are four kinds of variables in the SEM, namely the exogenous variables and endogenous variables, the latent variables (unobserved variables) and manifest variables (observed variables). The latent variable will be labeled in the ellipse, and the manifest variable will be labeled in the rectangle in SEM graphs. The SEM could analyze not only the empirical associations among different variables in the model, but also the statistical relationships between the latent variables and manifest variables simultaneously.

In this study, the exogenous variable was SES, while the endogenous variables were physical health and NLTC. Three latent variables were included in the current study, named SES, physical health, and NLTC. SES is measured by three manifest variables, namely education, income, and job before retire. Physical health was measured by four manifest variables: physical health last month, number of chronic diseases, pain/discomfort, and sick/injured last 2 weeks. NLTC was measured by four manifest variables, namely LTCN, ADL score, hearing loss and times of hospitalization.

The multi-group model invariance was determined before the discussion of the gender difference in the structural model of SEM. Only after the test was passed, indicating that the measurement invariance had been established, the comparison of the gender difference could be followed. The basic test strategy was to outline corresponds to model trimming when gradually restrict the initial unconstrained model by adding more constraints [47]. In this study, the partial measurement invariance was used to test the measurement invariance over multi-groups models in SEM. The change in root mean square error of approximation (RMSEA) was used to assess the measurement invariance for the comparison of the less restricted model with the more constrained model. With a sample of more than 300, the change of RMSEA (ΔRMSEA) is less than 0.015 implies that measurement invariance has been successfully established [48].

Evaluation of the goodness of fit calculates how a hypothetical model might be consistent with the empirical data. In the present study, generalized least squares estimation was employed to estimate the best-fitting model. The Chi-square test was used to assess the model’s fitness, as has been done in many other studies. However, when the sample size is large (as in this study), a non-significant Chi-square is rarely obtained [49,50] and the test is too dependent on sample size to be used in many conditions [51]. Thus, the Chi-square test was not adopted to evaluate the model fitness in the current study, although it was reported. Goodness of fit index (GFI), adjusted goodness of fit index (AGFI), RMSEA would be used as the fitness indexes and the models would be regarded to be well fitted when the *p* > 0.05, GFI > 0.90, AGFI > 0.90, and RMSEA < 0.05 in this study [52].

### 2.5. Ethical Considerations

Informed consent for the data collection and the use of the data was obtained from allsubjects. Medical ethics approval of this study was approved by the ethical committee of Shandong University (No. 20170110).

## 3. Results

### 3.1. Sample Characteristics

The information about the basic characteristics of the participants by LTCN is shown in Table 2. In total, 7070 subjects were finally included in the database. Of them, 2846 were male, accounting for 40.3% of the total (2846/7070), less than the number of female participants (*n* = 4224, 59.7% = 4224/7070). Regarding age, 1577, 2129, 1780 and 1584 subjects were in the 60–64, 65–69, 70–74, and >75 age group respectively. Concerning the LTCN of the participants, more females were found in the groups ”not needed“ (4046 females versus 2722 males) and ”needed” (178 females versus 124 males). Statistical differences between the male and female elderly based on LTCN were not found (*p* = 0.771). From the lower to higher age groups, 57, 69, 69 and 107 of the participants required LTC respectively, implying that LTC is needed more often by the older adults from the higher age group than the lower ones. A statistical difference was found between the four different age groups based on LTCN (*p* < 0.001). 

Table 3 showed the descriptive characteristics for each gender for selected variables. The most common education level was elementary school (2924 participants), while there were fairly low number of old adults were senior middle school or above (561). As for the annual income, it was evenly distributed in the categories apart from the highest income where there were fewer people earning this amount. 1685 of the older adults were in the income group of 0–2200 RMB per year, while 1414 participants were in the annual income groups of >21,400 RMB. The most common job before retire was farming/ forestry/fishing (5479 participants), followed by professional/ technical (442), transportation industry/ manual workers (383), and other jobs (274). While for the number of other four kinds of job before retire, they were all lower than 200. Statistical differences for education, annual income and job before retire between elderly men and women were found respectively (*p* < 0.001).

Most of the participants thought their physical health status over last month was fine, yet there were also 716 old adults rated their physical health as “fairly bad” and 82 of them rated as “very bad”. Regarding the total number of the chronic diseases, more than half of the subjects had at least one kind of chronic diseases, and 2200 of the participants had no chronic disease. 87 of the old adults had four or more kinds of chronic diseases. As for pain/discomfort, 3747 of the elderly participants had no pain/discomfort at all, 3146 had moderate pain/discomfort, and 177 were in a lot of pain/were uncomfortable. A total of 4509 of the older adults had been sick or injured during the last two weeks, while 2561 of them had not been sick or injured in the last two weeks. Statistical difference were shown between elderly men and women for physical health in the last month, number of chronic diseases, pain/discomfort, and being sick or injured during the last two weeks (*p* < 0.001).

A total of 302 older adults needed LTC, while the other 6768 elderly people did not need LTC. As for the ADL score, most of the participants had a fairly low score, since 5467 subjects had ADL score of 14, and only 169 of them scored 28 and over. Most of the elderly participants had not used the inpatient services durng the past 12 months, 996 had been hospitalized once, and 280 had used the inpatient services more than twice. Concerning the hearing loss, most of the elderly participants rated their hearing ability as normal, 1411 thought their hearing function had declined, and 237 were deaf and had lost their hearing function. Statistical differences were found between elderly men and women for ADL score and hearing loss, while no statistical differences between elderly men and women were found for LTCN and frequency of hospitalization. 

### 3.2. The Structural Equation Modelling Analysis

#### 3.2.1. Measurement Invariance across the Gender

Table 4 illustrates data collected on selected fitness indicators of measurement invariance and the fitness indexes of seven different models. The fitness indexes of the elderly men and elderly women were compared firstly to determine whether the variable “gender” is favorable for use in the group comparison. As shown in Table 4, the fitness indexes for the elderly men were GFI = 0.958, AGFI = 0.932, and RMSEA = 0.073 (M_1_), while for the elderly women, they were GFI = 0.962, AGFI = 0.939, RMSEA = 0.070 (M_2_). All fitness indexes showed values over 0.90 and very slight differences between the elderly men and women were found, indicating that we could furtherly compare the differences between the two genders. Although the RMSEA values of M_1_ and M_2_ were more than 0.05, these variables were mainly used to calculate the change of RMSEA, not to assess the model’s fitness.

The measurement invariance was then evaluated by the ΔRMSEA between M_3_ (Unconstrained Model); M_4_ (Measurement Weights Model), M_5_ (Structural Weights Model), M_6_ (Structural Covariance) and M_7_ (Structural Residuals). The Unconstrained Model restricts no coefficient in the model, the Measurement Weights Model assumes the indicator loadings for the corresponding construct are equal across the groups, and the Structural Weights Model constrains both the indicator loadings for the corresponding construct and the structural coefficients across the groups. The Structural Covariance Model assumes that the indicator loadings for the corresponding construct and the structural coefficients across the groups are equal and also that the covariance of the endogenous variables across the groups is equal. In the Structural Residuals Model, the indicator loadings, structural coefficients, covariance of the endogenous variables and variance of the exogenous variable across the groups are all assumed to be equal.

As stated before, when sample size is more than 300, a change in RMSEA (ΔRMSEA) of more than 0.015 would indicate non-invariance across groups [48]. As shown in Table 4, the ΔRMSEA is 0 between M_7_ and M_6_, 0 between M_6_ and M_5_, 0 between M_5_ and M_4_, 0.001 between M_4_ and M_3_, 0.020 between M_3_ and M_2_, 0.003 between M_2_ and M_1_. Considering the value of ΔRMSEA between M_2_ and M_3_ equaled 0.020, and all the other value of ΔRMSEA between different models was less than 0.015, the measurement invariance across gender was established. The comparison of the value between different variables across the groups of elderly men and elderly women individuals was then done.

#### 3.2.2. Model Fitness Indices

**Figure 1:**
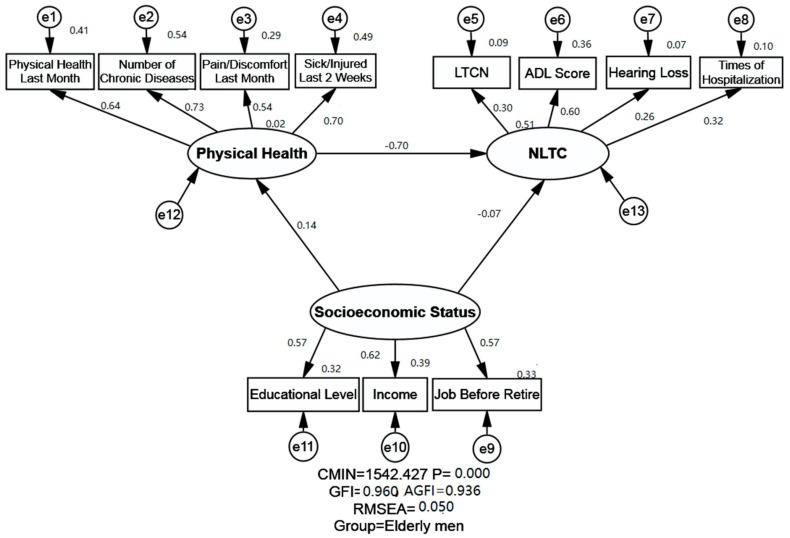
Structural equation modeling analysis of the association between socioeconomic status (SES), physical health and need for long-term care (NLTC) of the elderly men (*n* = 7070). Employing the cross-sectional data, association between SES, physical health and NLTC were analyzed. Arrows indicate the associations and directions between variables, double curved arrows indicate correlation between each factor. All parameter estimates were statistically significant (*p* < 0.001). Note: *χ*^2^ = Chi square; GFI = Goodness of Fit Index; AGFI = Adjusted Goodness of Fit Index; RMSEA = Root-mean Square Error of Approximation; LTCN= Long-term Care Needs; SES = Socioeconomic Status; NLTC = Need for Long-term Care; CMIN: Chi-square value in Amos is called CMIN.

**Figure 2:**
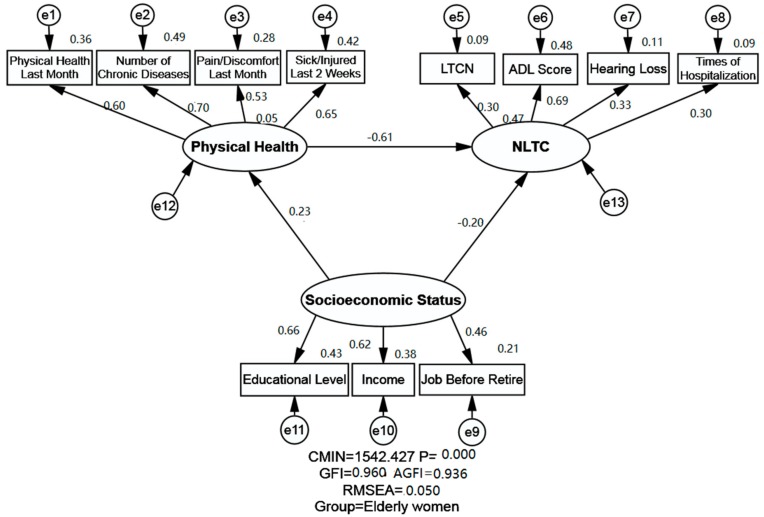
Structural equation modeling analysis of the association between SES, physical health and NLTC of the elderly women (*n* = 7070). Employing the cross-sectional data, association between SES, physical health and NLTC were analyzed. Arrows indicate the associations and directions between variables, double curved arrows indicate correlation between each factor. All parameter estimates were statistically significant (*p* < 0.001). Note: *χ*^2^ = Chi square; GFI = Goodness of Fit Index; AGFI = Adjusted Goodness of Fit Index; RMSEA = Root-mean Square Error of Approximation; LTCN = Long-term Care Needs; SES = Socioeconomic Status; NLTC = Need for Long-term Care; CMIN: Chi-square value in Amos is called CMIN.

The proposed model (Unconstrained Model) for the elderly men and elderly women is illustrated in Figure 1 and Figure 2, including three latent variables: SES, physical health and NLTC. The indexes of the model fitness were also presented in Table 4. The estimates of model fitness were similar for both genders: GFI = 0.960 > 0.90, AGFI = 0.936 > 0.90, RMSEA = 0.05. This indicates that the proposed model in this study fits the empirical data well both for the elderly men and elderly women. The Chi-square value is called CMIN in Amos, and was significant as shown in Table 4, with a *p* < 0.001. The CMIN indicates the fit of the model; however, it was not used to evaluate the fitness index in our study as many other researchers did, because it is very sensitive to the samples size.

### 3.3. Relationship between SES, Physical Health and NLTC by Using SEM

#### 3.3.1. Relationship between SES and NLTC

The relationship between SES, physical health and NLTC is presented in Table 5 and Figure 1 and Figure 2. SES and its indicators were shown to exert both direct and indirect effects (through physical health) on NLTC. A negative and direct effect of SES on NLTC was found both for the elderly men (standardized direct effects = −0.066) and elderly women (standardized direct effects = −0.197). Moreover, SES was shown to affect NLTC negatively and indirectly through physical health (standardized indirect effects = −0.096 for the elderly men and −0.139 for the elderly women). Ultimately, SES was found to exert a negative effect on NLTC for the elderly women (standardized total effects = −0.336 for elderly women), indicating that higher SES groups of elderly women generally have less chance of NLTC. It is noteworthy that the standardized effects of SES on NLTC showed no statistical significance for the elderly men, while the relationship between SES and NLTC was statistically significant for the elderly women.

#### 3.3.2. Relationship between Physical Health and NLTC

The relationship between physical health and NLTC was found to be negative for both elderly men and women, implying that better status of physical health of the elderly men and women is generally associated with less likelyhood of NLTC. Statistical significance was observed across genders, and the coefficient was slightly stronger for the elderly men (standardized total effects = −0.701 for the elderly men; standardized total effects = −0.614 for the elderly women).

#### 3.3.3. Relationship between SES and Physical Health

A positive and direct association between SES and physical health was observed both for the elderly men and elderly women, implying that a higher SES would indicate better physical health. Statistical significance was found across genders, and a slight difference was found between the elderly men and women, since the standardized total effect was 0.137 for the elderly men and 0.227 for the elderly women.

## 4. Discussion

### 4.1. Relationship between SES and NLTC

The negative relationship found in this study between SES and NLTC is similar to the results of research conducted in Canada [53], Japan [54], and Vietnam [13], which found the SES and its indicators were shown to be determinants of NLTC. To be specific, the association between education and NLTC was similar to the result of Hong’s study carried out among Korean elderly people [14]. While the relationship between income and NLTC was similar to that of Hamada’s research conducted among Japanese older adults [15]. The empirical results in this study may further explain why more unmet needs were reported by those who lived in the most deprived areas [55]. As for the gender difference, a negative effect of SES on NLTC was found among elderly women in the current study, while the association between SES and NLTC was not statistically significant. among the elderly men. Such a difference between the elderly men and elderly women on the association between SES and NLTC may due to the different characteristics/ composition of the research participants (such as there were more women than men in the database), or the cultural backgrounds on gender inequality (traditionally, men have a higher social position than women in Shandong Province, regardless of job, income, etc.). This result is different from Martikainen’s research conducted among Finnish elderly, which found the relationship between SES factors and NLTC was stronger for male elderly individuals than their female counterparts [56].

### 4.2. Relationship between Physical Health and NLTC

Physical health was found to be negatively related to NLTC in this study. This is similar to the research carried out among Japanese elderly [6] and Korean elderly [14], which also found a negative association between physical health and NLTC. Meanwhile, previous researches conducted in the United States of America [8,57] and the United Kingdom [9] also found that having comorbid physical health problems (number of chronic diseases, pain/discomfort) was associated with a higher probability of NLTC. All of these studies show us a well-known social phenomenon—as the physical health status of the elderly decreases, their NLTC generally increases.

### 4.3. Relationship between SES and Physical Health

The prevalence of chronic diseases and pain/discomfort were found to be higher among the female elderly individuals in this study. This is similar to the research completed by Schiotz, which found that physical and mental health comorbidities wer more prevalent in women than men [24]. Our result is also similar to that found in Nagamine’s study, which showed that a clear income gradient for diabetes prevalence, which was higher for the Japanese elderly women than the Japanese elderly men [27].

The relationship found between SES and physical health in this study is consistent with the results of previous studies, which generally showed a positive association between the socioeconomic characteristics of the individuals and their physical health [58]. Specifically, lower SES is generally associated with worse physical health outcomes, such as higher rates of long-term mortality among stroke survivors [28], low WHOQOL scores [22], and a lower likelihood of improving functional ability among those with severe disabilities [59]. Our study was identical to one study among the Japanese elderly which also found a positive association between SES and physical health [6]. As for the gender difference, the result of our study is same as a previous study which found that the effect of SES on physical health was similar among the elderly men and women [25], but slightly higher among the elderly women.

### 4.4. Relationship between SES, Physical Health and NLTC

Besides the current study, many previous studies have also investigated the relationship between the health status and SES. The results consistently presented a positive association between health and SES, which means a higher SES generally predicts better health [37,60,61,62]. The effect of SES on health is so profound that elderly people with a relatively higher childhood SES have significantly better well-being than their lower childhood SES counterparts [63]. It is widely acknowledged that the health differences of the individuals are rooted in the fundamental conditions of social context and experience [18]. As the basic background information of the individuals, SES and its effect on health could also partially explained by other related macro and micro factors. Ethnicity [64], perceived control [65], physical activity [66], lifestyle, environmental and social factors [67], and ecological factors [68] have also been found to be determinants/mediators of the association between SES and health. Specifically, the different levels of SES could affect health through exposure to different capabilities/opportunities to access health services, social policy, social support and social capital, occupational hazards and environmental pathogens, cumulative effects of stress and differences in health risk behaviors [69].

Higher life expectancies, more fragile pension and social security systems around the world, and less extended families and traditional systems of support for the elderly all make the studies on the association between SES and the health status of the elderly important [70]. In the current study, the empirical association between SES, physical health and NLTC in Chinese elderly was explored by using SEM. The findings of our study and previous research could be explained as follows: older adults from different SES levels live in different communities and environments, they have different life style and different levels of access to health services. All of these factors influence physical health status. As the physical health of lower SES elderly individuals tends to decline first, their NLTC emerges and gradually increases.

### 4.5. Implications

As the population ages, the LTC systems are becoming more and more important at the local and national levels. The empirical association between SES, physical health and NLTC clarified in the current study have implications for the development of LTC system, or on lowering or delaying the need for LTC. Firstly, the relationship of SES with both NLTC and physical health indicates that policy makers should attach great importance to the advancement of an individual’s SES. Various social and political initiatives should be implemented to lower social inequality, since it is hard to achieve from the individual level, especially for vulnerable elderly people and families. Specifically, the government could take measures to increasing the education, income and social status of the elderly, as well as the accessibility and affordability of health care. The income of seniors could be enhanced by increasing their retirement allowance and refunding a percentage of their health care expenditure. Regarding the improvement of education, lifelong learning policies could be formed and promoted by the government on both local and national levels. Secondly, the negative relationship between physical health and NLTC implies the importance of improving the physical health status of the elderly and its further effect on lowering their NLTC. Health policy makers and practitioners should formulate strategies to improve the health status of the population, based on individuals’ demographic/socioeconomic information, cultural backgrounds and related resources.

Shandong Province has taken measures to enhance the SES of vulnerable individuals and the elderly in many aspects. Under the guidance of the National Poverty Alleviation and Rural Revitalization Strategy, Shandong Province now pays more attention to low-income groups and has taken various measures to increase the income and improve the life quality of the rural population through employment, education and health care. For example, in Zibo City of Shandong Province, the “first village doctor” system was initiated in 2017 [71]. Young doctors from the municipal medical and health units were selected to serve as "first village doctors", who mainly provide health care services, help to alleviate the risk of poverty due to health care spending, and provide operational guidance and support to the village doctor. In this way, the elderly in the rural area are able to enjoy high quality medical care services, reducing the chances of having a low SES because of health care expenditure. Furthermore, Shandong Province also formulated the “Working program for establishing a national model province on the integration of medical and nursing care” in 2018. Under the guidance of this plan, Shandong Province will establish a relatively perfect standard, management system and professional personnel training system for the integration of medical and nursing care by the end of 2020. All medical institutions will provide a green channel for the elderly, and the family doctor will cover all elderly at this point. It is worth noting that the fees paid for the family doctors of the elderly will be shared by the local government and the elderly [72]. In this way, the accessibility to the medical and nursing care will be improved, while the economic burden of the elderly will be reduced. These health care related policies implemented in Shandong Province would be helpful on improving the equity of accessibility and usage of medical services, not only between the elderly from the urban and rural areas, but also between the elderly men and elderly women. To be specific, these measures on the more reasonable distribution of the medical care resources would reduce the disparity in physical health status and LTC services caused by differences on gender and SES.

It is also noteworthy that as the population ages both the national and global levels, more and more formal and informal caregivers are being required, and special attention should also be given to the physical health condition of these caregivers. One study conducted among Welsh adults found that one in five of them reported caring for a sick, disabled, or frail person. Of those, caregivers who had back pain experienced slight physical health and emotional problems, whereas caregivers without back pain experienced worse physical health and emotional problems that could even disrupt normal life [68,73]. In consideration of caregivers’ unique position to influence older residents to participate in regular physical activity and thereby enhancing their physical health status [74], the reduction of the physical, mental and economic burdens of caregivers is an important issue for policy makers. Arai and Zarit’s study suggested that the LTC insurance scheme in Japan appears to be an effective measure for alleviating the burden of family caregivers [75], as it is a universal health care system that is beneficial for all related individuals and groups [24].

### 4.6. Limitation and Future Research

The limitations of the current study are as follows. Firstly, the need for formal long-term care services is traditionally understood as a person/environment interaction variable that reflects an individual’s physical and cognitive functioning and/or complex medical needs, as well as the availability of household amenities and informal support to cope with these barriers to daily living. However, not all the related variables about NLTC are concluded in our database following the description above (such as informal assistance measure available). Only four variables (long-term care needs, ADL Score, Times of Hospitalization, Hearing Loss) were used to evaluate the NLTC. The rationality for using these four indicators to assess the NLTC needs more discussion. Secondly, four indicators were used to assess physical health: “How was your physical health status last month (physical health last month)?”, “How many chronic diseases did you have during the last six months (number of chronic diseases)?”, “Did you have pain or discomfort last month (pain/discomfort)?”, and “Were you sick or injured during the last two weeks (sick/injured last 2 weeks)”. Yet there is adequate evidence that the accumulation of diagnoses is not a great indicator of health status—someone can have a moderate number of diagnoses but still have a long life, and someone else can have just one diagnosis and lots of barriers to survival. More information about physical health, such as stroke, obesity, fracture and other physical problems, were not included in this study due to limitation of time, financial and other related resources. Thirdly, the ADL score and hearing loss were used as indicators to evaluate the NLTC in this study. The authors initially wanted to use the ADL score and hearing loss as indicators for physical health. However, the ADL score, together with hearing loss, were deemed to be the indicators of NLTC by the exploratory factor analysis. The appropriateness of the use of these two indicators for NLTC should be furtherly considered. Fourth, LTCN was a nominal observed variable to assess the latent variable (NLTC) in the SEM analysis of this study. The variable used in the SEM analysis are generally required to be ordinal ones. However, the national LTC system has not been established so far in China, which made the assessment of NLTC of the elderly difficult compared with those countries that have already formed a national LTC system. Some developed countries, such as Japan, South Korea and Germany, have already established national LTC systems, and clarified the corresponding caring level of each older adults joined the system. This would make it easy for the researchers to determine the NLTC of the seniors. In the current study, we assessed the NLTC of the elderly by asking them “do you need LTC”, with the answer given as “yes/no”. More discussion on the difference in NLTC evaluation between self-report and functional assessment by medical professionals is needed.

Association between SES and health might result from a mixture of lifestyle, biological, ecological, environmental and social factors, not just one single cause. The effect of lifestyle (such as dietary, sleeping, smoking) on health is widely acknowledged. Researches on the relationship between SES, lifestyle, physical health and NLTC are needed in the future. Moreover, the World Health Organization (WHO) defines health as a state of complete physical, mental and social well-being and not merely the absence of disease or infirmity [76]. Besides physical health, there are also mental health and social well-being aspects. Further researches on their relationship with SES and NLTC are also needed.

## 5. Conclusions

The present study explored the empirical relationship between SES, physical health and the NLTC of the older adults in Shandong Province, China. Physical health was shown to exert a strong and negative effect on the NLTC for both genders, and a slightly stronger effect was found among the elderly men. SES was found to be significantly and negatively related to the NLTC among the elderly women, while no statistical significance was found for the association between SES and NLTC for the elderly men. A significant and positive association between SES and physical health was observed among the elderly men and women, with a slightly stronger effect among the elderly women.

## Figures and Tables

**Table 1:** Measurements of the variables in this study.

Variables	Definition	Measurements	Options
Socioeconomic Status	A broad concept that compositely assess an individual’s economic and sociological position in relation to others	Education	(1) Illiteracy, (2) Graduate from Elementary School, (3) Graduate from Junior Middle School, (4) Graduate from Senior Middle School and above
		Annual income	(1) less than 2200 RMB (USD = 326), (2) from 2201 to 3300 RMB (USD = 489), (3) from 3301 to 6200 RMB (USD = 919), (4) from 6201 to 21400 RMB (USD = 3170), (5) more than 21400 RMB (USD = 3170).
		Job before retire	(1) Professionals/ technical, (2) Leaders of government/ public institution, (3) Clerks or staff, (4) Businessman/ commercial servants, (5) Farming/ forestry/ fishing industry, (6) Transportation industry/ manual workers, (7) Privately/ individually-owned business, (8) Other jobs.
Physical Health	The efficient functioning of the body and its systems, and includes physical capacity to perform tasks and physical fitness.	How was your physical health status last month (physical health last month)?	(1) Very bad (2) Fairly bad (3) Moderately (4) Fairly good (5) Very good
		How many chronic diseases did you have during the last six months (number of chronic diseases)?	(1) 0 (2) 1 (3) 2 (4) 3 (5) ≥4
		Did you pain or discomfort last month (pain/discomfort)?	(1) None (2) Moderate (3) Very painful/uncomfortable
		Were you sick or injured during the last two weeks (sick/injured last 2 weeks)	(1) No (2) Yes
Need for long-term care	A person/environment interaction variable reflecting the individual’s physical and cognitive functioning and/or complex medical needs and the availability of household amenities and informal supports to cope with these barriers to daily living.	Long-term care needs	(1) Not needed (2) Needed
		ADL Score	(1) 14 (2) 15 (3) 16 (4) 17 (5) 18–27 (6) ≥28
		Times of Hospitalization	(1) 0 (2) 1 (3) ≥2
		Hearing Loss	(1) Normal (2) Hearing function declined (3) Deaf

ADL: Activities of Daily Living.

**Table 2:** Characteristic of Participants by LTCN.

Variable	LTCN						
	Need (*n* = 302)		No Need (*n* = 6768)		Total (*n* = 7070)		χ^2^ Test
	*n*	%	*n*	%	*n*	%	
Gender							
Male	124	4.4%	2722	95.6%	2846	100.0%	0.085 *p* = 0.771
Female	178	4.2%	4046	95.8%	4224	100.0%	
Age							
60–64	57	3.6%	1520	96.4%	1577	100.0%	31.767 *p* < 0.001
65–69	69	3.2%	2060	96.8%	2129	100.0%	
70–74	69	3.9%	1711	96.1%	1780	100.0%	
>75	107	6.8%	1477	93.2%	1584	100.0%	

LTCN = long-term care needs.

**Table 3:** Descriptive Characteristic of Participants by Gender.

Variable	Gender						χ^2^ Test
	Men (*n* = 2846)		Women (*n* = 4224)		Total (*n* = 7070)		
	*n*	%	*n*	%	*n*	%	
Educational Level (graduated from)							
Illiteracy	491	21.6%	1779	78.4%	2270	100.0%	561.142 *p* < 0.001
Elementary School	1294	44.3%	1630	55.7%	2924	100.0%	
Junior Middle School	718	54.6%	597	45.4%	1315	100.0%	
Senior Middle School and above	343	61.1%	218	38.9%	561	100.0%	
Income (RMB per year)							
0–2200	505	30.0%	1180	70.0%	1685	100.0%	117.736 *p* < 0.001
2201–3300	439	38.1%	714	61.9%	1153	100.0%	
3301–6200	607	43.2%	797	56.8%	1404	100.0%	
6201–21,400	652	46.1%	762	53.9%	1414	100.0%	
>21,400	643	45.5%	771	54.5%	1414	100.0%	
Job Before Retire							
Professionals/technical	243	55.0%	199	45.0%	442	100.0%	121.792 *p* < 0.001
Leaders of government/public institution	111	61.7%	69	38.3%	180	100.0%	
Clerks or staff	87	53.0%	77	47.0%	164	100.0%	
Businessman/ commercial servants	28	24.1%	88	75.9%	116	100.0%	
Farming/forestry/fishing industry	2107	38.5%	3372	61.5%	5479	100.0%	
Transportation industry/manual workers	174	45.4%	209	54.6%	383	100.0%	
Privately/individually-owned business	14	43.8%	18	56.3%	32	100.0%	
Other jobs	82	29.9%	192	70.1%	274	100.0%	
Physical Health Last Month							
Very bad	39	47.6%	43	52.4%	82	100.0%	42.948 *p* < 0.001
Fairly bad	275	38.4%	441	61.6%	716	100.0%	
Moderate	578	34.9%	1079	65.1%	1657	100.0%	
Fairly good	1126	40.3%	1666	59.7%	2792	100.0%	
Very good	828	45.4%	995	54.6%	1823	100.0%	
Number of Chronic Diseases							
0	1022	46.5%	1178	53.5%	2200	100.0%	
1	1015	40.0%	1522	60.0%	2537	100.0%	74.537
2	602	36.6%	1045	63.4%	1647	100.0%	*p* < 0.001
3	161	29.3%	389	70.7%	550	100.0%	
≥4	46	33.8%	90	66.2%	136	100.0%	
Pain/Discomfort							
None	1685	45.0%	2062	55.0%	3747	100.0%	
Moderate	1098	34.9%	2048	65.1%	3146	100.0%	73.716
Very painful/uncomfortable	63	35.6%	114	64.4%	177	100.0%	*p* < 0.001
Sick/Injured Last 2 Weeks							
No	188	46.4%	1373	53.6%	2561	100.0%	62.813
Yes	1658	36.8%	2851	63.2%	4509	100.0%	*p* < 0.001
LTCN							
Not needed	2722	40.2%	4046	59.8%	6768	100.0%	0.085 *p* = 0.771
Needed	124	41.1%	178	58.9%	302	100.0%	
ADL Score							
14	2274	41.6%	3193	58.4%	5467	100.0%	25.090 *p* < 0.001
15	171	35.5%	311	64.5%	482	100.0%	
16	67	30.2%	155	69.8%	222	100.0%	
17	70	34.1%	135	65.9%	205	100.0%	
18–27	191	36.4%	334	63.6%	525	100.0%	
≥28	73	43.2%	96	56.8%	169	100.0%	
Times of Hospitalization							
0	2332	40.2%	3462	59.8%	5794	100.0%	0.001
1	401	40.3%	595	59.7%	996	100.0%	*p* = 0.999
≥2	113	40.4%	167	59.6%	280	100.0%	
Hearing Loss							
Normal	2087	38.5%	3335	61.5%	5422	100.0%	33.453 *p* < 0.001
Hearing function declined	637	45.1%	774	54.9%	1411	100.0%	
Deaf	122	51.5%	115	48.5%	237	100.0%	

LTCN = long-term care needs, ADL = activities of daily life.

**Table 4:** Multi-group Model Invariance Test.

Model	χ^2^	d.f.	χ^2^/d.f.	GFI	AGFI	RMSEA	ΔRMSEA
M_1_	661.239 ***	41	16.128	0.958	0.932	0.073	-
M_2_	881.184 ***	41	21.492	0.962	0.939	0.070	0.003
M_3_	1542.427 ***	82	18.810	0.960	0.936	0.050	0.020
M_4_	1618.308 ***	90	17.981	0.958	0.939	0.049	0.001
M_5_	1626.370 ***	93	17.488	0.958	0.941	0.048	0
M_6_	1626.832 ***	94	17.307	0.958	0.941	0.048	0
M_7_	1632.518 ***	96	17.005	0.958	0.941	0.048	0

M_1_ = Elderly Men; M_2_ = Elderly Women; M_3_ = Unconstrained; M_4_ = Measurement Weights; M_5_ = Structural Weights; M_6_ = Structural Covariance; M_7_ = Structural Residuals; *** *p* < 0.001. d.f.: degrees of freedom; GFI: Goodness of Fit Index; AGFI: Adjusted Goodness of Fit Index; RMSEA: Root-mean Square Error of Approximation.

**Table 5:** Standardized Effects between SES, Physical Health and NLTC by Gender.

Variable	Direct		Indirect		Total	
	Men	Women	Men	Women	Men	Women
SES→NLTC	−0.066	−0.197 ***	−0.096	−0.139 ***	−0.162	−0.336 ***
Physical Health→NLTC	−0.701 ***	−0.614 ***	-	-	−0.701 ***	−0.614 ***
SES→Physical Health	0.137 ***	0.227 ***	-	-	0.137 ***	0.227 ***

Notes: SES = socioeconomic status, NLTC = need for long-term care, *** *p* < 0.001.
